# Institutionalization of assistive health technology in India: establishment of a national centre within the IIT-AIIMS ecosystem

**DOI:** 10.3389/fresc.2026.1879168

**Published:** 2026-09-09

**Authors:** Sunanda Deb, Rinila Das, Ravinder Singh

**Affiliations:** 1 Division of Non Communicable Diseases, Indian Council of Medical Research, New Delhi, India; 2 Division of Delivery and Implementation Research, Indian Council of Medical Research, New Delhi, India

**Keywords:** assistive technology (AT), functional impairments, health policy, India, rehabilitation systems

## Abstract

**Background:**

Assistive technology (AT) is now recognised as a major element of universal health coverage and rehabilitation systems. Over 2.5 billion individuals around the world need at least one assistive product and this number is expected to increase significantly by 2050. Despite international guidance through the World Health Organization Global Cooperation on Assistive Technology (GATE) and Rehabilitation 2030 initiatives, many low- and middle-income countries (LMICs) lack institutional mechanisms to integrate AT into mainstream public health systems. In India AT provision has historically been fragmented and welfare-orientated.

**Objective:**

This paper provides a detail of the development of AT systems in India which subsequently led to the institutional development, governance architecture, and functional design of the National Centre of Assistive Health Technology (NCAHT) in India and discusses its potential implications for strengthening AT systems and policy implementation.

**Methods:**

This paper employs a process-based institutional case study design. It explains the original institutional process undertaken by ICMR to establish NCAHT between 2017 and 2023. Primary data comprised ICMR concept notes, expert committee minutes, inter-ministerial consultation records, and formal institutional proposals generated during the establishment process.

**Evolution of NCAHT:**

India participation in WHO-GATE initiative and international rehabilitation system alignment offered a preparatory strategy that resulted in the NCAHT, 2022 in India. NCAHT is a project funded by the ICMR, and operates as a collaboration between the AlIMS Delhi & IIT Delhi. The Centre operates at the national and international levels in line with WHO 5P framework, ICMR-NLEAP, BIS-ICMR-Standards, WHO Rehabilitation-2030, integrated care for older people (ICOPE), Ear & Hearing Care (EHC), SPECS-2030 ecosystem. NCAHT has established institutional functions across twelve domains, collectively aimed at operationalizing AT as an integral component of health and rehabilitation systems rather than a welfare-driven intervention as discussed in this paper.

**Conclusions:**

NCAHT provides a systems-level framework which can be used to enhance access to AT within health and rehabilitation services. Its development presents lessons applicable to the development of AT systems in the low- and middle-income countries based on the coordinated governance, evidence-based validation, and multi-sector cooperation.

## 1 Introduction

Assistive technology (AT) is becoming an essential part of the rehabilitation system and universal health coverage, as it allows people with functional impairments to realize independence and participation as well as have improved quality of life. The World Health Organization (WHO) estimates that more than 2.5 billion people worldwide require at least one assistive product, with demand expected to double by 2050 due to population ageing, increasing prevalence of non-communicable diseases and injuries (1). Globally, significant gaps remain in the availability, affordability, and systematic delivery of assistive technologies, particularly in low- and middle-income countries (LMICs). The Lancet Commission on Global Health and Disability has documented that over 80% of persons with disabilities reside in LMICs, where AT coverage rates remain critically low due to fragmented policy environments, weak supply chains, and limited workforce capacity (2) Access to safe, affordable and appropriate assistive products is therefore central to universal health coverage (UHC), healthy ageing and disability inclusion (3). Recognising this growing need, the WHO launched the Global Cooperation on Assistive Technology (GATE) initiative in 2014 and subsequently articulated the Rehabilitation 2030 agenda, which emphasises integration of rehabilitation and assistive products within health systems (4, 5). The WHO GATE framework promotes a systems-based approach for strengthening AT services. It is structured around five key pillars-People, Policy, Products, Provision, and Personnel, collectively known as the 5P model, which together aim to support the development of accessible, sustainable, and person-centered AT systems. The GATE initiative of WHO is drawing together people who share the GATE vision: a world where AT is universally accessible to everyone, everywhere (4). In spite of these international initiatives, most LMICs still use a fragmented, welfare-based delivery system of AT. Some countries have adopted community-based rehabilitation (CBR) frameworks, while others have built centralized national AT programmes integrated within the health system (1, 6). These systems are usually poorly coordinated in terms of governance, regulatory controls, systematic clinical validation and long term funding (6). The delivery of AT in India has been based on social welfare programmes or disability-oriented programmes with limited integration into mainstream health research, regulatory framework and digital health infrastructure. To address these structural gaps, the Indian Council of Medical Research (ICMR) launched a multi-phase reform undertaking between, which resulted in the establishment of the National Centre of Assistive Health Technology (NCAHT).

The paper places NCAHT in the context of international rehabilitation and AT, examines its institutional design and operations. This paper describes the institutional development, governance architecture, and operational framework of NCAHT, situating it within the international rehabilitation and AT systems context. We propose that India's AT ecosystem demonstrates a coordinated, scalable model for integrating research, validation, manufacturing, workforce development, and policy within the health system. Such an approach may serve as a replicable model for other low- and middle-income countries (LMICs) seeking to institutionalize AT beyond fragmented or welfare-driven schemes.

## 2 Methodology

### 2.1 Study design

This paper employs a process-based institutional case study design to describe the establishment of the NCAHT in India, drawing on primary documentation generated during the period 2017–2023. Documents were identified through institutional archives and included if they directly pertained to the establishment, governance, or operational design of NCAHT. Given the close institutional involvement of the authors in the conception, funding, governance, and documentation of NCAHT, potential sources of bias are explicitly acknowledged. To enhance transparency and minimize bias, findings are grounded in primary institutional records and cross-referenced against published WHO frameworks, with a clear distinction maintained between planned activities, established governance structures, ongoing processes, and anticipated outcomes. Patient or public involvement was not applicable to this institutional establishment process.

### 2.2 Primary data sources

The evidence base for this paper comprises primary institutional records generated during the establishment of NCAHT, including: (i) ICMR concept notes and internal divisional records (2017–2022); (ii) minutes of expert committee meetings convened by ICMR, including the National Expert Group meeting (October 2019) and National Expert Committee meetings (January 2020); (iii) inter-ministerial and multi-stakeholder consultation records, including the deliberations of the Ministry of Health and Family Welfare (MoHFW), NITI Aayog, and the ICMR Governing and Executive Councils (August 2020); (iv) formal proposals, concept documents, and letters of agreement between ICMR, AIIMS New Delhi, and IIT Delhi; (v) partnership and stakeholder meeting records involving agencies such as BIRAC, MeitY, TIDE-DST, DEBEL, BIS, C-DAC, and WHO-India; and (vi) ICMR's participation records in WHO-GATE consultations, including the GREAT Summit (2017) and SEAR regional consultation (December 2017).

### 2.3 Data analysis

Data were analysed using a qualitative descriptive approach. Information extracted from the included documents was organised and synthesised using the WHO GATE 5P framework (People, Policy, Products, Provision, and Personnel). This analytical framework is used as a structured approach for examining the institutional development and operational components of NCAHT. Data extraction focused on key governance decisions, institutional milestones, implementation processes, stakeholder roles, partnerships, and functional domains. The findings were then grouped into thematic categories aligned with the five domains of the GATE framework to describe the establishment, governance, implementation, and strategic priorities of NCAHT.

## 3 Evolution of NCAHT

### 3.1 Sequence of activities

In 2017, the WHO hosted the first international program on AT, where close to 200 stakeholders addressed issues, opportunities, and innovations of AT research, education, policy, products, personnel, provision, and use. The program highlighted the significance of the 5Ps model and the worldwide effect of enhancing the access to assistive technologies (ATs) to individuals with functional impairments (7). ICMR held a regional consultation with SEAR countries in December 2017. Among the suggestions of this consultation was to have R&D (Research and Development) infrastructure in India so as to enhance access to AT. The goals of the programs like Health for All (HFA), Universal Health cover (UHC), Sustainable Development Goals (SDG's), United Nations Convention on the Rights of Persons with Disabilities (UNCRPD), are to enhance inclusivity and find local solutions to the problems facing the country (8). A major conference on the subject of the National Centre for Assistive Technology was organized on May 13, 2019, at the ICMR Headquarters in New Delhi. During that meeting a decision was made that ATs would be led in India and South-East Asia region by ICMR. One of the key outcomes of the consultation was intent to establish a Centre for Advanced Research and Excellence in Assistive Technologies (CARE-AT) through collaboration between the All India Institute of Medical Sciences (AIIMS), New Delhi, and the Indian Institute of Technology (IIT) Delhi (9–11). The proposed centre aimed to strengthen innovation and research in ATs through collaboration with leading international institutions such as University College London (UCL) and the Department for International Development (DFID).

Based on discussions from the first meeting of the WHO adhoc Advisory Group of Experts on AT and the Global Report on Effective Access to Assistive Technology (GReAT) attended by ICMR, a partnership meeting was held on 5 August 2019 to discuss the establishment of a NCAHT. The proposal was subsequently endorsed during a National Expert Group meeting on 4 October 2019. Following this, AIIMS New Delhi and IIT Delhi agreed to provide dedicated space within their campuses, along with existing infrastructure and manpower, to support the proposed centre.

A detailed proposal for NCAHT was presented to the National Expert Committee on 7 January 2020 and later submitted to the Minister of Health and Family Welfare (MoHFW). Based on the Minister's recommendations, a stakeholder meeting involving key institutions and agencies including IIT, AIIMS, SIB-AIIMS, BIRAC, MeitY, TIDE-DST, DEBEL, SAMER, C-DAC, C-MET, BARC, BIS, and WHO-India—was held on 19 June 2020 to further deliberate on the proposal. The proposal was subsequently presented to the Executive Council (19 August 2020) and the Governing Council (20 August 2020) of ICMR for consideration. The centre is proposed to be set up under the direct stewardship of ICMR, New Delhi, within the campuses of AIIMS–New Delhi and IIT–Delhi with the focus to engage other AIIMS, IITs as well as other key stakeholders. The Minister of Health and Family Welfare (HFM) proposed that other government agencies that deal with AT be engaged to provide their contributions and determine how they can collaborate and develop NCAHT centers in India (9–11). In 2022, the ICMR officially inaugurated the NCAHT in partnership with AIIMS-Delhi and IIT-Delhi. The AIIMS center is being set up as of December 2022 and will initiate operations early 2023.The National Center for Assistive Health Technologies (NCAHT-IITM), established on June 17, 2023, at IIT Madras, is a premier initiative by the Indian Council of Medical Research (ICMR) and implemented by the TTK Center for Rehabilitation Research and Device Development (R2D2).This center was established to meet the growing demand of the innovation, design and development of ATs to people with functional impairment in India (8–10). The institutional design intentionally integrated: Health research governance (ICMR); Clinical validation infrastructure (AIIMS); Engineering and innovation ecosystems (IITs). This tri-sectoral architecture sought to overcome fragmentation between innovation, validation, policy and standard development.

### 3.2 Objectives of NCAHT

The ICMR-NCAHT has the following objectives:

1. Conducting a comprehensive and ongoing analysis of the landscape of functional impairments in relation to ATs, covering issues from multiple aspects such as the burden of disease preventable by ATs, penetration of ATs, and barriers to access, unmet needs, and value chain gaps.
2. Creating a repository of knowledge regarding ATs to assist various stakeholders, including doctors, engineers, patients, NGOs, welfare organizations, innovators, and governments, in making informed decisions.
3. Fostering innovation and establishing a framework for the validation of new and existing technologies, as well as the implementation and scaling of novel ATs.
4. Liaising with the National Health Authority (NHA) to ensure the expeditious inclusion of ATs in the list of reimbursable interventions.
5. Bringing all stakeholders together under a common cause of rehabilitation, chiefly through ATs.
6. Enhancing national capacity in training for innovations in novel ATs, functional impairment research, and public policy briefs.
7. Conducting public policy landscaping and making relevant policy recommendations to improve access to and utilization of rehabilitative ATs.
8. Collaborating with industry and the Bureau of Indian Standards (BIS) to facilitate the creation of relevant standards in a way that supports local manufacturing, ensures patient safety, and maintains a level playing field for all.

### 3.3 Roles and responsibilities of partners

ICMR will be responsible for conducting landscape studies and providing overall coordination, planning, project monitoring, and evaluation. It will facilitate collaborations through agreements and Memoranda of Understanding (MoUs) and ensure effective liaison among various stakeholders. Additionally, ICMR will oversee fund management, provide intellectual property management services, and support technology transfer along with post-transfer monitoring to ensure successful implementation and sustainability of developed technologies.

AIIMS, New Delhi would play a key role in clinical immersion and the clinical validation of the developed technologies. The institute would also focus on fellowship management and fellow recruitment; and also assist in the formation and development of start-ups that will enhance innovation and translation of research findings into practical applications in healthcare.

IIT Delhi would concentrate on research and technology advancement to the area of visual impairment. The institute would also join the effort with other IITs in solving technological solutions in other fields of disability, hence encouraging the multidisciplinary research and innovations in AT development.

The overall project will be monitored and evaluated by a governance board along with other designated committees, as outlined in the appraisal note, to ensure effective oversight, accountability, and progress of the project.

### 3.4 Major activities in accordance with the WHO 5P framework-

**Figure 1:**
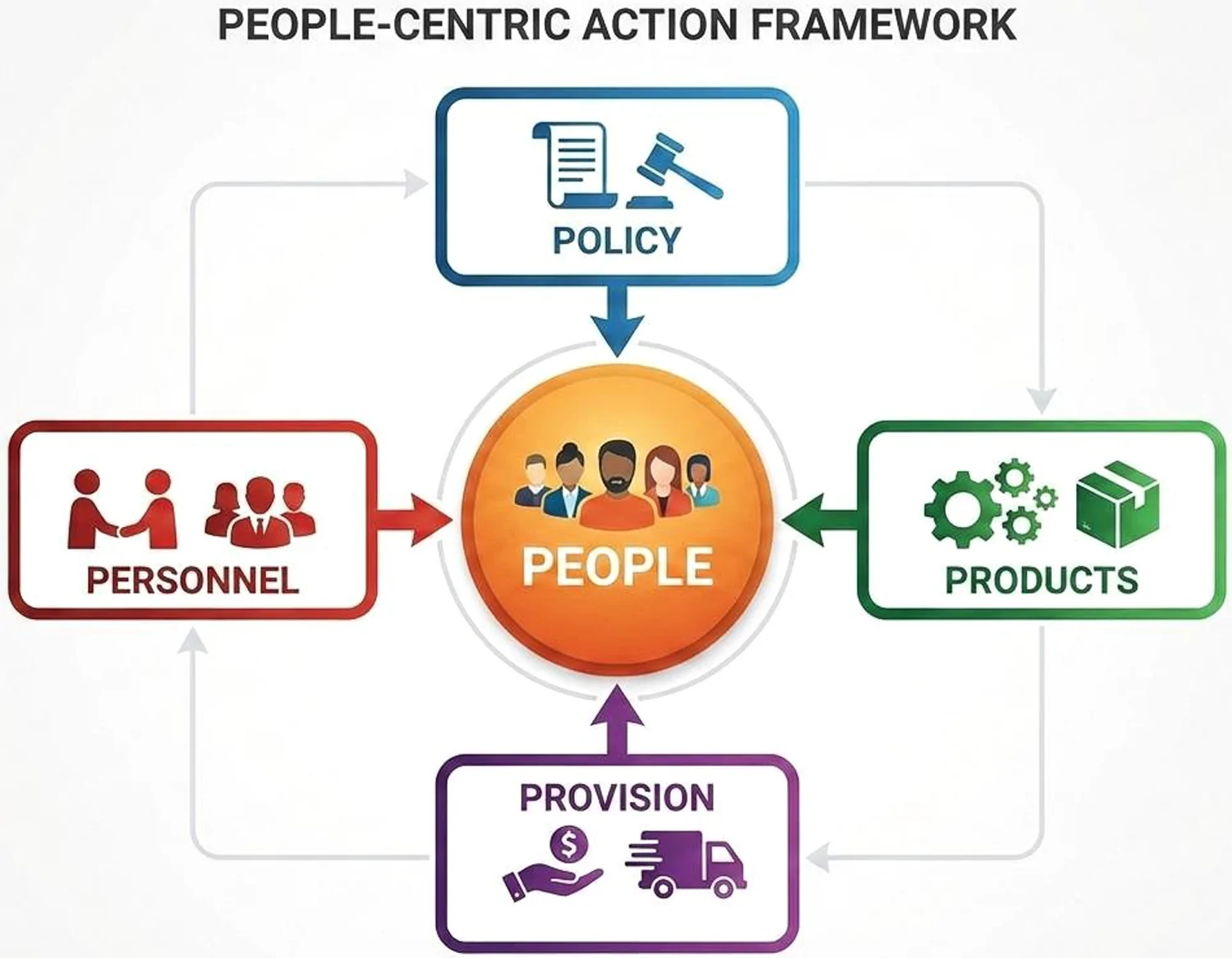
World Health Organization, Global Cooperation on Assistive Technology (GATE) 5P framework (4).

The activities are organized in terms of the WHO-GATE 5P framework that is a comprehensive way of enhancing access to AT in the world (4). The framework consists of five essential elements, namely, People, Policy, Products, Provision, and Personnel. All these elements are important in making sure that AT is delivered to those who are most in need and that its value is achieved. NCAHT uses the WHO GATE 5P framework into the planning and implementation of assistive health technology in India, as discussed below (Figure 1).

1. **People**: This element highlights that AT systems must be designed around the needs, preferences, and rights of users. It is about creating awareness, increasing social and professional understanding of the significance of AT, and making the voices and experiences of the users be at the forefront of the design and execution of ATs (4). The Centre emphasises user-centred design and rights-based approaches aligned with the UN Convention on the Rights of Persons with Disabilities (UNCRPD) which calls for accessible environments, assistive devices, rehabilitation services, and research to support persons with disabilities (11–13). User engagement is incorporated throughout the innovation cycle-from problem identification and prototype development to usability testing and validation
2. **Policy**: One of the areas of interest of GATE activities is the collaboration with governments, policymakers, and international organizations to formulate and execute policies that facilitate access to AT. This involves lobbying for laws, regulations and policies that will enable inclusion of AT within the healthcare systems and also ensuring that mechanisms to support financially accessible healthcare to all exist (4). NCAHT contributes to this by conducting policy research, stakeholder consultations, and standards development for ATs (13). The centre collaborates with national agencies and policymakers, provides technical inputs to the Ministry of Health and Family Welfare, NITI Aayog and BIS to support inclusion of assistive products within UHC frameworks and national standards systems. It also supports integration with national digital health initiatives such as the Ayushman Bharat Digital Mission (14), which promotes interoperable digital health records and standardized health data systems. Linking AT services with these digital platforms can strengthen care coordination, referral tracking, and monitoring of rehabilitation services.
3. **Products**: Availability and affordability of AT products are important because of the fact that persons with functional impairments will be able to access the devices they are in need of. The GATE activities have focused on the improvement of the development and quality of AT products to be affordable, flexible and efficient to satisfy the different needs of different users. This includes support in innovation, promotion of development of user-orientated products, and ensuring that the products are well supplied (4). NCAHT helps in research, design, and development of new and context-relevant assistive products by interdisciplinary teams of clinicians, engineers, designers and rehabilitation workers. The centre encourages the indigenous innovation and assists in the assessment of the devices and refinement of the devices that are already in place so that they are of good quality and they suit the needs of the users. These efforts can be used to fill the disparity in the supply of low-cost and locally applicable ATs (11).
4. **Provision**: It is necessary to ensure that ATs are availed in an effective and efficient manner. GATE emphasizes the need to establish the distribution, maintenance and repair systems and services of AT products. This includes the establishment of delivery systems to the underserved areas and the general improvement of the entire services delivery that allow individuals to easily access the AT when they need (4). NCAHT strives to reinforce models of service delivery through incorporation of ATs in clinical workflow and rehabilitation services. To achieve safety, usability and performance before large-scale adoption, a structured clinical validation framework is developed in collaboration with tertiary care institutions (11).
5. **Personnel**: This dimension emphasizes the need for a trained workforce capable of prescribing, fitting, and maintaining ATs. This will involve healthcare professionals, technicians, and service providers who are conversant with AT, how to prescribe it, and how to make sure it is used correctly (4). NCAHT addresses this through capacity-building initiatives, training programs, and interdisciplinary fellowships involving clinicians, engineers, rehabilitation professionals, and innovators. These training programmes aim to develop expertise in AT innovation, service delivery, and implementation research, thereby strengthening national capacity for AT development and provision.

### 3.5 Functional domains of NCAHT

Through multiple partnerships, NCAHT functions as a distributed national network for AT research, innovation, and clinical validation, strengthening the evidence base and implementation ecosystem for ATs in India. NCAHT operates through twelve interrelated functional domains that collectively support the AT value chain. These domains research, innovation, capacity building, policy inputs, advocacy, documentation, resource generation, mentorship, incubation, training, dissemination, and collaboration. This represents a systems-level institutional framework for advancing AT and rehabilitation services in the country (1, 5). Rather than functioning as isolated activities, these twelve functional domains operate as interconnected components of a national AT system. Research generates evidence; innovation translates it into context-specific products; clinical validation ensures safety and performance; policy inputs institutionalize standards; and collaboration with industry supports manufacturing and scale. Together, these domains address structural gaps across the AT value chain from design and testing to service delivery and regulation (Table 1).

1. **Research**: NCAHT has institutionalized AT research in India by coordinating multicentric and interdisciplinary studies addressing unmet needs related to mobility, sensory, communication, and educational impairments. This research-based solution addresses historic gaps in the evidence to address the integration of AT in public health and rehabilitation systems
2. **Innovation**: NCAHT has facilitated the creation and translation of various ATs by facilitating multidisciplinary grouping of clinicians, engineers, doctors, designers, and end users. By linking academic institutions, healthcare facilities, and industry partners, NCAHT strengthens the innovation pipeline from conceptualization to field-ready solutions. Particular emphasis is placed on affordability, usability, and contextual relevance of ATs
3. **Capacity Building**: NCAHT has also provided a coordinated national structure for clinical validation of ATs for the first time in India, implemented through AIIMS and other collaborating institutions. These validation activities assess safety, usability, performance, and user acceptability prior to large-scale adoption, thereby improving confidence among users, developers, healthcare providers and regulators.
4. **Policy Inputs**: NCAHT has made technical and evidence-based contributions to national policymaking processes with the Ministry of Health and Family Welfare, NITI Aayog and BIS. Examples of notable contributions are the standardisation of assistive products, compatibility with the National List of Essential Assistive Products (NLEAP) and the inclusion of AT within primary healthcare and digital health systems like the Ayushman Bharat Digital Mission.
5. **Advocacy:** NCAHT also plays an important role in advocating for the recognition of AT as an essential component of health and rehabilitation systems, rather than solely a welfare intervention. The Centre promotes the inclusion of AT in universal health coverage, life-course rehabilitation, and rights-based frameworks based on the recommendations of the United Nations Convention on the Rights of Persons with Disabilities (UNCRPD).
6. **Documentation:** The NCAHT Centre has enhanced the documentation of the country by coming up with repositories of proven technologies, clinical evidence, technical reports, policy briefs, and best-practice guidelines. This is an established record that facilitates transparency, institutional memory and evidence-based decisions in various sectors.
7. **Resource Generation**: NCAHT contributes to mobilizing financial and institutional resources through government funding mechanisms, inter-institutional partnerships, and industry collaborations. Aligning AT initiatives with national health priorities enhances sustainability and facilitates scaling of innovations.
8. **Mentorship:** NCAHT mentorship structure is offered to early-career researchers, innovators, clinicians and engineers involved in ATs and rehabilitation. This mentorship ecosystem offers interdisciplinary skills and leadership to overcome workforce discontinuities emphasized by national and global models of rehabilitation.
9. **Incubation**: NCAHT facilitates incubation and translation of proven ATs with the help of linkages to innovation ecosystems and industry partners. This incorporates regulation pathway facilitation, clinical feedback, and market preparedness, which allows innovations to move beyond pilot processes to scalable deployment.
10. **Training:** NCAHT has also introduced training programmes, workshops and professional reorientation programs among the clinicians, rehabilitation professionals, engineers and frontline health workers. Such initiatives follow the WHO Rehabilitation 2030 agenda, which focuses on integrating service delivery, competency based training, and life-course rehabilitation and AT provision.
11. **Dissemination:** The Centre spreads knowledge and promotes adoption of assistive technologies through a broad range of dissemination and engagement activities. These include national and regional workshops, podcasts, exhibitions, and technology demonstrations that engage diverse audiences including clinicians, engineers, policymakers, and the general public. Dissemination increases awareness of proven technologies, standards, and models of service delivery and helps health systems and implementing agencies to adopt them.
12. **Collaboration:** NCAHT is an organization that serves as a coordination platform in the country to connect ministries, academic institutes, standards bodies, industry partners, and user groups. Industry collaboration fosters indigenous production, quality control, and commercialization, as well as makes them affordable and aligned to the users.

**Table 1:** Implementation Status of functional domains of the NCAHT.

Sl. No.	Functional domain	Key activities	Implementation status
1	Research	Multicentric research, needs assessment, product development, clinical and translational research	Operational
2	Innovation	Product design, prototype development, user-centred design	Operational
3	Capacity Building	Clinical validation framework, institutional strengthening, Technical training	Operational
4	Policy Inputs	Technical guidance, policy recommendations, standards development, support for national AT initiatives	Initiated
5	Advocacy	Stakeholder engagement, awareness generation, promotion of AT within health and rehabilitation systems	Operational
6	Documentation	Development of technical reports, policy briefs, evidence repositories, knowledge resources	Operational
7	Resource Generation	Government funding, institutional partnerships, industry collaboration, resource mobilization	Operational
8	Mentorship	Mentoring researchers, clinicians, engineers, innovators	Operational
9	Incubation	Technology transfer, commercialization support, regulatory facilitation, industry engagement	Initiated
10	Training	Workshops, professional training programmes, skill development	Operational
11	Dissemination	Scientific publications, conferences, exhibitions, stakeholder consultations, outreach activities	Operational
12	Collaboration	Partnerships among government, International bodies (WHO), academia, healthcare institutions, industry, standards bodies, and user organizations	Operational

Altogether, all these twelve functions make NCAHT a backbone of the Indian AT and rehabilitation ecosystem. NCAHT has also enabled access to affordable and reliable assistive products to support mobility, education, communication and independent living of people with functional impairments by promoting indigenous innovation, evidence-based policy, and coordinated governance. The institutional model offers a potentially replicable framework for other low- and middle-income countries seeking to strengthen AT systems within health care.

As of the study period, the NCAHT network comprises four operational Centres established across India, each contributing complementary expertise in AT research, development, clinical validation, and translation (Table 2). Through this collaborative institutional model, a portfolio of 37 AT products has been initiated and is currently progressing through different stages of the technology development pathway, representing multiple Technology Readiness Levels (TRLs) (Table 3). Of the 37 products, four have been successfully developed and officially launched (Table 4).

**Table 2:** National centre for assistive health technologies (NCAHT) centres established in India.

Sl. No.	NCAHT CENTERS	Date of establishment	Role	Website links
1	NCAHT—Indian Institute of Technology Delhi	2022 (ICMR-funded initiative; products launched in June 2024)	Research & innovation;knowledge generation;	https://www.ncaht.in/
			National coordination; innovation for visual impairment; technology validation; technology transfer; training and capacity building, experience zone	https://home.iitd.ac.in/show.php?id=238&in_sections=Press
2	NCAHT—All India Institute of Medical Sciences, New Delhi	December 2022 (operations initiated in early 2023)	AT Innovation and related training; landscape research; industry support & standards research; policy research and advocacy; ecosystem strengthening; clinical integration and research; clinical validation of ATs	https://www.ncahtaiims.in/
3	NCAHT—Indian Institute of Technology Madras	June 2023	AT Innovation and research; AT experience zone; prototype development; rehabilitation engineering; user–developer interface; training and demonstration centre.	https://ncaht.iitm.ac.in/
4	NCAHT—National Institute of Speech and Hearing (NISH)	2022	AT Innovation and research; AT experience zone; service delivery for hearing, speech, communication, and other ATs	https://www.nish.ac.in/others/faculty-staff/assistive-technology-and-innovation
				https://www.ncaht.in/

**Table 3:** List of AT products being developed under NCAHT.

Sl. No.	Name	Description
1	Ultra Lightweight Wheelchair	A highly portable and durable wheelchair designed for effortless mobility with minimal weight.
2	Power Assist for wheelchairs	A motorized attachment that enhances manual wheelchair propulsion, reducing user fatigue.
3	Pool Lift	A mechanical assistive device enabling safe and easy transfer of individuals with disabilities into swimming pools.
4	Saathi Walker	For children with Cerebral Palsy.
5	Boccia Ramp	An assistive ramp for athletes with severe physical disabilities to accurately launch Boccia balls during the game.
6	Stair climber	A mechanized device that enables wheelchair users to navigate staircases safely and independently.
7	OmniBed	Convertible Multi- Configuration Patient-Care Platform
8	Vayu—Low-Cost Mechanical Insufflation–Exsufflation (Cough-Assist) Device	Respiratory assistive device designed to improve airway clearance by mechanically assisting coughing in individuals with neuromuscular disorders, spinal cord injuries, and other conditions associated with weak cough
9	Auto-Cath—Automated Secretion Suction system	Auto-Cath automates tracheal suctioning by sensing secretion buildup and performing clearance at optimal times, reducing missed events, nurse workload, and airway complications.
10	Pediatric Neck and Trunk Control Device	An assistive positioning device designed to provide head, neck, and trunk support, stability, and improving posture, in children with neuromotor impairment
11	Neuro Vest	AI-Powered Smart Wearable for Therapy for Pediatric Patients with Neuromotor Impairments
12	Redesigned Eye Drop Bottle	Self-Stabilizing Ocular Dispenser with Nose-Based Alignment.A user-friendly device that enables individuals, especially those with limited dexterity, to accurately apply eye drops independently.
13	Neuro Vest	AI-Powered Smart Wearable for Therapy for Pediatric Patients with Neuromotor Impairments
14	Hearing Alert	A device that notifies individuals with hearing impairments about important sounds using visual or vibration alerts.
15	CP Chair Generation Platform	A customizable design platform for developing posture-supportive chairs for individuals with cerebral palsy.
16	Contextual AAC	An Augmentative and Alternative Communication (AAC) system that provides context-aware assistance for non-verbal individuals.
17	Single Button Communicator Switch	A simple, accessible switch enabling individuals with motor impairments to communicate using a single-button interface.
18	Vestibular Vertical Perception test	A diagnostic tool for assessing balance and spatial orientation issues related to vestibular disorders.
19	Wheelchair Pressure Mapping System	A system that monitors and analyzes pressure distribution to prevent pressure sores in wheelchair users.
20	Vestibular AI based Autism Detection	An AI-powered diagnostic tool that uses vestibular responses to assist in early autism detection.
21	Flash card generation platform	A digital platform enabling teachers to create flashcards with images and text downloadable as PDFs.
22	Shape Scape	An interactive tactile learning tool designed to help individuals with functional impairments explore geometric shapes, spatial concepts, and creative design.
23	Accessible STEM Kit	A comprehensive kit enabling students with functional impairments to engage in STEM education.
24	High Quality White Cane	A durable and ergonomically designed cane providing enhanced mobility and safety for individuals with visual impairments.
25	Smart Cane 2.0	An advanced electronic mobility aid that detects obstacles and provides haptic feedback to individuals with visual impairments.
26	TacRead	A tactile reading device designed to improve Braille literacy and accessibility for individuals with visual impairments.
27	Children Cane	Lightweight and adjustable mobility cane tailored for children with visual impairments to enhance independent navigation.
28	Tacto Sketch Board	A tactile drawing and writing board that allows individuals with visual impairments to create and explore graphics.
29	Accessible Science Experiments	A collection of adapted science experiments ensuring hands-on learning for students with functional impairments.
30	Accessible Coding Addons	Assistive tools that enable individuals with functional impairments to participate in coding and programming activities.
31	AI tool for Entrance Detection	A smart system using AI to identify and assist in locating doorways and entry points for individuals with visual impairments.
32	AI tools for accessible image descriptions	AI-powered technology that generates detailed image descriptions, improving digital accessibility for individuals with visual impairments.
33	Accessible coding with Quorum	A programming platform designed to be accessible for individuals with visual impairments.
34	Magic Platform	An interactive and accessible learning platform for inclusive education and skill development.
35	Touching Cultures	A multisensory educational tool that enables individuals with visual impairments to explore cultural artifacts through touch.
36	Reconfigurable Audio Announcer	A customizable audio-based navigation and announcement system for public spaces to assist individuals with functional impairments.
37	Accessible Periodic Table	A tactile and audio-enabled periodic table designed for learners with visual impairments to explore chemical elements.

**Table 4:** List of assistive technology products launched under the NCAHT .

Sl. No	Product name	Lead NCAHT centre	Launch date	Target users	Official website/source
1	Shapescapes (a geometry learning kit)	NCAHT IIT Delhi	26 June 2024	Students with visual impairment	https://home.iitd.ac.in/show.php?id=238&in_sections=Press
					https://www.biospectrumindia.com/news/101/24841/national-centre-for-assistive-health-technologies-at-iit-delhi-launches-new-products.html
2	High-Quality White Cane	NCAHT IIT Delhi	26 June 2024	Persons with visual impairment	https://home.iitd.ac.in/show.php?id=238&in_sections=Press
					https://www.biospectrumindia.com/news/101/24841/national-centre-for-assistive-health-technologies-at-iit-delhi-launches-new-products.html
3	Accessible STEM Education Kit	NCAHT IIT Delhi	26 June 2024	Blind and low-vision students	https://home.iitd.ac.in/show.php?id=238&in_sections=Press
					https://www.biospectrumindia.com/news/101/24841/national-centre-for-assistive-health-technologies-at-iit-delhi-launches-new-products.html
4	YD One—Ultra-Lightweight Active Wheelchair	NCAHT IIT Madras (R2D2)	16 July 2025	Persons with mobility impairments	IIT Madras/R2D2 (Indian Institute of Technology Madras)

Apart from NCAHT centres there are 5 Collaborating Centre for Assistive Health Technology (CCAHT) established. CCHATs are designated to work in collaboration with the NCAHT to strengthen India's AT ecosystem. CCAHTs support the development, clinical validation, testing, capacity building, research, innovation, and dissemination of ATs while contributing expertise in their respective domains. The 5 CCAHT centres are AIIMS Bhatinda, AIIMS Bhopal, AIIMS Jodhpur, AIIMS Kalyani, AIIMS Bibinagar.

## 4 Discussion

This paper describes the evolution of AT systems in India and the institutional development that led to the establishment of NCAHT. In contrast to project-based strategies that are fragmented, as witnessed in most LMICs, NCAHT is an institutionalised process that brings together innovation, clinical validation, standards development and policy coordination through a single national framework in India. This aligns with global evidence highlighting the growing demand for ATs and the persistent gaps in access, particularly in low- and middle-income countries (LMICs). Access to ATs remains limited and dispersed across welfare programmes in many LMICs due to fragmented governance, weak regulatory frameworks, and shortages of trained personnel, which limits integration into mainstream health systems (15, 16). The WHO health system building blocks framework (17) and Atun et al.'s work on integration of health interventions (18) both suggest that isolated, project-based approaches are insufficient for achieving scale and equity in AT delivery—a conclusion supported by experience in multiple LMIC contexts (17–19).

Within the Indian context, earlier research has highlighted similar structural challenges. Studies on AT services in India describe fragmented delivery systems, limited service planning, inadequate production capacity, and insufficient national-level coordination among stakeholders involved in AT provision (20, 21). The institutional architecture of NCAHT attempts to address these limitations by creating a centralized but distributed platform that links health research institutions, clinical centres, engineering ecosystems, industry partners, and policy bodies. Under the rehabilitation systems viewpoint, NCAHT implements some of the main principles of the Rehabilitation 2030 agenda, which include integrated service delivery, workforce development, and data-informed planning (5). The inter-sectoral governance mode between health, engineering, standards bodies, and industry provides a transferable template to the other LMICs to institutionalise AT outside the social welfare paradigms. Nevertheless, long-term effect will require sustained funding, increasing regional centers, and integrating with digital health platform, to facilitate scale and equity.

The National Conference on Sustainable Provision of Assistive Technology (NCONSPAT-23) further highlights the importance of developing coordinated national systems for AT in India. The conference brought together policymakers, clinicians, engineers, industry representatives, and disability organisations to outline strategies for strengthening access to AT beyond the National List of Essential Assistive Products (NLEAP). Some key recommendations were the integration of rehabilitation into health systems, the empowerment of quality and regulatory frameworks, the growth of the AT workforce by competency-based training, the encouragement of innovation ecosystems and local manufacturing, and the uptake of user-centred design strategies. These priorities closely align with the institutional functions of the NCAHT, reinforcing its role as a national platform for advancing AT systems in India (22).

From an implementation science perspective (23), the NCAHT model incorporates several features associated with successful scale-up of health innovations in LMICs. Milat et al.'s scalability framework identifies multi-sector partnerships, policy alignment, and resource generation as critical enablers of sustainable scale (24). Similarly, Barker et al.'s review of AT scale-up in LMICs identifies national coordination bodies and policy integration as the most consistent determinants of expanded AT access (25). India's model differs from many high-income country frameworks in several ways. First, it focuses on lean, resource-conscious engineering that fits well in a resource-starved setting. Second, it integrates public institutions (ICMR, AIIMS), engineering institutes (IITs), and NISH, and industry partners within a unified governance structure. Third, local manufacturing ecosystems are based on affordability and scalability. Lastly, integration on the district level in the public health system will offer a platform to coverage at the population level. However, long-term sustainability will depend on sustained financing, expansion of regional nodes, measurable documentation, and deeper integration with digital health systems to ensure scale and equity. This paper is limited by its reliance on institutional documents and policy records rather than independent impact evaluations, and therefore primarily reflects a systems and governance perspective.

## 5 Conclusion

Through integration of global systems with national interests, NCAHT offers a sustainable way of enhancing access to effective, affordable, and safe assistive products for functionally impaired people in India. Lessons of Indian experience can be used by other LMICs to empower rehabilitation systems and promote inclusive health technologies by means of institutional leadership and evidence-based governance.

Looking ahead, the strategic vision of NCAHT includes expanding regional centres, strengthening national validation and data systems, and leveraging emerging technologies such as digital health platforms, robotics, and artificial intelligence to enhance rehabilitation services. Continued policy integration and international collaboration may further strengthen India's AT ecosystem. While ongoing evaluation will be necessary to assess long-term impact, the institutional model offers useful insights for other low- and middle-income countries seeking to develop coordinated and sustainable AT systems.

### 5.1 Limitations

The findings are based primarily on institutional records and policy documents and have not been independently externally validated or benchmarked against other national AT systems. As NCAHT is an evolving national initiative, this paper does not evaluate service utilization, patient or health system outcomes, or include the perspectives of AT users and caregivers. Future independent evaluations incorporating implementation, user, and health system outcomes will be valuable in assessing the effectiveness, scalability, and transferability of the NCAHT model.

## Data Availability

The raw data supporting the conclusions of this article will be made available by the authors, without undue reservation.
